# *Aspergillus*-Derived Galactosaminogalactan Triggers Complement Activation on Human Platelets

**DOI:** 10.3389/fimmu.2020.550827

**Published:** 2020-10-06

**Authors:** Hemalata Deshmukh, Cornelia Speth, Donald C. Sheppard, Magdalena Neurauter, Reinhard Würzner, Cornelia Lass-Flörl, Günter Rambach

**Affiliations:** ^1^Institute of Hygiene and Medical Microbiology, Medical University of Innsbruck, Innsbruck, Austria; ^2^Christian Doppler Laboratory for Invasive Fungal Infections, Innsbruck, Austria; ^3^Department of Microbiology and Immunology, McGill University, Montréal, QC, Canada

**Keywords:** aspergillosis, galactosaminogalactan, platelets, complement, innate immunity, invasive fungal infections

## Abstract

Invasive fungal infections caused by *Aspergillus* (*A*.) and Mucorales species still represent life-threatening diseases in immunocompromised individuals, and deeper knowledge about fungal interactions with elements of innate immunity, such as complement and platelets, appears essential for optimized therapy. Previous studies showed that galactosaminogalactan secreted by *A. fumigatus* and *A. flavus* is deposited on platelets, thereby inducing their activation. Since the altered platelet surface is a putative trigger for complement activation, we aimed to study the interplay of platelets with complement in the presence of fungal GAG. Culture supernatants (SN) of *A. fumigatus* and *A. flavus* both induced not only GAG deposition but also subsequent deposition of complement C3 fragments on the platelet surface. The SN of a Δ*uge3* mutant of *A. fumigatus*, which is unable to synthesize GAG, did not induce complement deposition on platelets, nor did the SN of other *Aspergillus* species and all tested Mucorales. Detailed analysis revealed that GAG deposition itself triggered the complement cascade rather than the GAG-induced phosphatidylserine exposure. The lectin pathway of complement could be shown to be crucially involved in this process. GAG-induced complement activation on the platelet surface was revealed to trigger processes that might contribute to the pathogenesis of invasive aspergillosis by *A. fumigatus* or *A. flavus*. Both pro-inflammatory anaphylatoxins C3a and C5a arose when platelets were incubated with SN of these fungal species; these processes might favor excessive inflammation after fungal infection. Furthermore, platelets were stimulated to shed microparticles, which are also known to harbor pro-inflammatory and pro-coagulant properties. Not only did early processes of the complement cascade proceed on platelets, but also the formation of the terminal complement C5b-9 complex was detected on platelets after incubation with fungal SN. Subsequently, reduced viability of the platelets could be shown, which might contribute to the lowered platelet numbers found in infected patients. In summary, fungal GAG initiates an interplay between complement and platelets that can be supposed to contribute to excessive inflammation, thrombocytopenia, and thrombosis, which are important hallmarks of fatal invasive mycoses.

## Introduction

Although invasive fungal infections are rare in immunocompetent individuals, they remain a significant cause of morbidity and mortality in immunocompromised patients. Medical progress leads to improved survival of chronically immunosuppressed patients, but the reverse of the medal is an increasing number of patients at risk for fungal infections (1, 2).

Lung infections due to *Aspergillus* species are caused by inhalation of ubiquitously present airborne conidia, with *Aspergillus fumigatus* and *Aspergillus flavus* as predominant human pathogenic species (3). Invasion of *Aspergillus* hyphae into the pulmonary arterioles initiates hematogenic dissemination, as it occurs in approximately one-third of patients with pulmonary aspergillosis. Consequences include thrombosis, hemorrhagic infarctions, and involvement of distant organs (4, 5). Mortality rates up to 40–50% are reported in patients with acute leukemia and recipients of hematopoietic stem cell transplantation suffering from invasive aspergillosis (6).

Various secreted fungal components have been described as relevant virulence factors, thus essentially contributing to the pathogenesis of invasive infections. One of these components is the heteropolysaccharide galactosaminogalactan (GAG), which is secreted by actively growing hyphae of *A. fumigatus* and *A. flavus* (7). Other *Aspergillus* species or Mucorales do not produce GAG. Fungal GAG fulfills multiple roles in the interaction between pathogen and host [reviewed in (8)]. Many of these roles concern an interference with the immune system, including neutrophils, cytokine production, and T-cell response. Recent results revealed that GAG acts as an activator of platelets (9). This aspect is of particular interest, since platelets show a broad spectrum of immunological competences and thus might at least partly replace other immune weapons that are down-modulated by GAG (8, 10, 11). Platelets, acaryote cell fragments derived by megakaryocytes, are endowed with a comprehensive panel of pathogen detection molecules. When pathogen-associated molecular patterns are sensed, platelets can act both autonomously to fight the invaders, and they can collaborate with other soluble or cellular immune elements in a complex network (10, 12).

The complement system is a key collaboration partner of platelets within the immune network. Complement represents a multi-component cascade made of about 30 soluble and membrane-bound factors, regulators, and receptors. Similar to platelets, complement represents a fast-acting part of the innate immunity with a comprehensive spectrum of pathogen sensors and a versatile armamentarium to fight against invaders. Classical pathway, alternative pathway, and lectin pathway all differ in protein composition and their mechanisms to recognize foreign structures. However, they all converge at the level of the central complement factor C3; processing of C3 constitutes the starter reaction of the common terminal pathway. Numerous complement activation products are central immunological effector molecules. Opsonins derived from C3 cleavage act as natural adjuvants, supporting effective phagocytosis and contributing to a proper B-cell reaction (13, 14). The anaphylatoxins C3a and C5a modulate many immunological processes, mainly in a pro-inflammatory manner and via binding to their corresponding receptors that are expressed on a broad range of cell types (15). The C5b-9 complex, also called membrane attack complex (MAC) or terminal complement complex (TCC), is the final product of the terminal pathway. As a cytolytic effector, it forms a pore in the membrane of pathogens or targeted cells and thus induces osmolysis. Besides this function, C5b-9 can trigger intracellular signaling cascades and cell activation [reviewed in (16)].

Our previous studies showed that secreted GAG activates platelets and is deposited on their surface (9). Both platelet activation, with exposition of phosphatidylserine from the inner to the outer side of the plasma membrane, and deposition of foreign GAG polysaccharide onto the platelet membrane might stimulate the complement system to recognize these platelets as foreign. Putative consequences would be their opsonization, generation of pro-inflammatory complement products, and platelet loss by direct lysis or by stimulation of complement receptor-bearing phagocytes. Both features, excessive inflammation, and thrombocytopenia, are characteristics of invasive fungal infections. For that reason, we tested the hypothesis that GAG-triggered platelet modifications lead to complement activation in invasive fungal infections with platelet loss and pro-inflammatory reactions as putative respective consequences.

## Materials and Methods

### Antibodies, Chemicals, and Media

Most of the antibodies were from BioLegend (San Diego, CA, USA), C3c antibody was from Dako (Glostrup, Denmark), and C5b-9 antibody was ordered from Hycult Biotech (Uden, The Netherlands). Soybean agglutinin (SBA) lectin was from Vector labs (Burlingame, CA, USA), and the synthetic peptides sunflower MASP inhibitor peptides SFMI-1 and SFMI-2 were from Metabion (Planegg, Germany). Human C3a and C5a ELISA kits were purchased from BD Biosciences (San Diego, CA, USA), calcein blue AM was from Thermo-Fisher Scientific (Waltham, MA, USA), and thrombin was from Sigma-Aldrich (St. Louis, MO, USA).

RPMI1640 medium (R6504, Sigma-Aldrich) was supplemented with 19.8 g/L glucose and 34.5 g/L MOPS and adjusted to pH 7.0 with NaOH. Sabouraud glucose medium came from BD Diagnostic Systems (Heidelberg, Germany). Supplemented minimal medium (SUP) was prepared with yeast extract (Roth, Karlsruhe, Germany), supplemented with glucose, NH_4_Cl, KH_2_PO_4_, K_2_HPO_4_ (Roth), and MgSO_4_ (Merck, Darmstadt, Germany) (17).

### Fungal Isolates and Preparation of Fungal Culture Supernatants

Clinical isolates of most *Aspergillus* and all mucormycete species were obtained from patients with proven invasive aspergillosis or mucormycosis with various underlying diseases; three *A. versicolor* isolates were environmental strains. The *A. fumigatus* mutant strain Δ*uge3* was constructed from the parental strain *AF293* (18); this deletion of *uge3* completely abolishes GAG synthesis. This mutant was described to show normal growth and morphology, but lower adherence to a variety of substrates including pulmonary epithelia cells, and attenuated virulence in mouse models (19). The Δ*uge3* strain was routinely included in the experiments as negative control. A detailed table of all used strains of *Aspergillus* spp. and Mucorales species is shown elsewhere (9).

*Aspergillus* spp. isolates were cultured on Sabouraud glucose agar (SAB), and mucormycetes on Supplemented Minimal Medium (SUP) agar, for 3–5 days at 37°C, until sporulation was clearly visible. *A. versicolor* was grown at 30°C, and *Mucor* spp. was cultured at 28°C. Spores were rinsed off with phosphate buffered saline (PBS, supplemented with 0.05% Tween-20) and filtered through a 40-μm and a 10-μm cell strainer (BD Biosciences). The conidial suspension was counted in a hemocytometer, adjusted to a concentration of 1 × 10^8^/ml in PBS and kept at 4°C. To obtain fungal culture supernatants (SN), 1 × 10^4^ spores of *Aspergillus* or mucormycetes strains were inoculated in 500 μl of RPMI medium with high glucose content and incubated at 37°C for 2 days. Fungal elements were removed by centrifugation, and SN was obtained by filtration using Spin-X filters (Corning Life Sciences, Corning, NY, USA). The fungal SN was used freshly or kept frozen at −20°C for further use.

### Preparation of Human Platelets

All studies were approved by the local ethics committee (Nr. AN5170 328/4.1 342/5.2). Whole blood from healthy volunteers with informed consent was collected in a trisodium citrate blood collection system (Sarstedt, Nürnbrecht, Germany) with avoidance of shear stress; for one experiment, the anticoagulant citrate-theophylline-adenosine-dipyridamole (CTAD; Greiner Bio-One, Kremsmünster, Austria) was used instead of citrate. Platelet-rich plasma (PRP) was obtained by centrifugation of blood at 135 × *g* for 15 min (slow deceleration) at room temperature (RT). A concentration of 2 × 10^5^ platelets/μl in RPMI medium was adjusted after counting in a hemocytometer. All steps were performed in low-binding tubes with inert surfaces to avoid spontaneous platelet activation.

Platelet concentrates were provided from the local Department of Immunology and Blood Transfusion. Platelets were collected from healthy blood donors and prepared by thrombocytapheresis with Amicus cell separator (Baxter, Deerfield, IL, USA). The platelet number was routinely determined with a hemocytometer and adjusted to a concentration of 1.2–1.4 × 10^6^/μl in RPMI medium.

To obtain human serum, whole blood of healthy volunteers was collected without anticoagulants and centrifuged for 15 min at 1,500 × *g* at RT. Serum from at least five donors was pooled and stored at −80°C for further use.

### Analysis of Platelet Parameters by Flow Cytometry and Confocal Microscopy

Platelets (PRP or platelet concentrates) were pre-incubated with RPMI medium, 0.1 IU/ml thrombin, or SN (20% v/v, unless otherwise stated) of different fungal isolates for 30 min at 37°C, followed by addition of 40% v/v (unless otherwise stated) human serum (or PBS control) as complement source. For analysis of C3c deposition, incubation was continued for further 30 min. To detect C5b-9 formation and deposition on platelets, the further incubation time was 120 min.

Samples were fixed with 1% formalin for 30 min (RT), washed with PBS by centrifugation at 1,100 × *g*, and incubated for 30 min (RT) with fluorescence-labeled specific antibodies. To quantify GAG deposition, platelets were stained with FITC-labeled SBA lectin, as previously described elsewhere (9). All samples were analyzed by flow cytometry (FACSVerse^TM^, BD Diagnostics). Platelets were gated by forward and side scatter and by the use of a labeled antibody against the platelet marker CD41 (BioLegend). Secretion of α-granules during platelet activation results in surface exposure of CD62P (P-selectin), which was detected by a specific fluorescence-labeled antibody (BioLegend). Complement deposition was measured by the use of a C3c antibody, which detects both the C3c fragment of the C3 protein and the C3c part of C3b. The C5b-9 antibody binds to a C9 neoantigen of the TCC; this neoantigen is absent from native C9 prior to complex formation. To assess budding of platelet-derived microparticles, the platelets were incubated in the presence or absence of fungal SN and human serum for 90 min as described above and fixated with 1% formalin. The microparticles were analyzed by flow cytometry by gating of the adequate size and binding of labeled CD41 antibody. Furthermore, microparticles were visualized by confocal microscopy.

For confocal microscopy, the samples were prepared as described above and, after washing, embedded in liquid mounting medium (Mowiol, Sigma-Aldrich), followed by visualization employing a confocal microscope (PerkinElmer).

### Determination of Platelet Viability by Calcein Blue

Calcein blue AM, a cell-permeant esterase substrate, measures both enzymatic activity and cell-membrane integrity. Calcein blue AM is only weakly fluorescent. Upon cleavage by intracellular esterases, this tracer becomes polar and stays intracellular when the membrane is intact. Furthermore, its fluorescence intensity increases and its fluorescence spectra shift to longer excitation/emission maxima. Calcein blue AM was used according to the manufacturer's instructions. Briefly, 10 μM calcein stain was added to each sample along with the addition of serum or buffer to the platelets that were pre-incubated with medium or different concentrations of fungal SN. After fixation in 1% formalin and washing with PBS, the calcein signal was quantified by flow cytometry.

### C3a and C5a ELISA

The anaphylatoxins C3a and C5a are generated upon complement activation and rapidly cleaved to the C3a-desArg and C5a-desArg form, respectively, by the endogenous serum carboxypeptidase N enzyme. The commercially available kits are solid phase sandwich ELISAs and quantify C3a-desArg and C5a-desArg in biological samples. The kits were used according to the manufacturer's instructions (BD Biosciences). Briefly, platelets were pre-incubated for 30 min with medium, 0.1 IU/ml thrombin, or 20% fungal SN derived from different species, followed by addition of serum as complement source for another 30 min. Platelets were removed by centrifugation, and the SN samples were transferred to the pre-coated ELISA wells. After washing the wells with PBS, the respective biotinylated detection antibodies and the substrate were added and the color signals were quantified in an ELISA reader at 450 nm.

### Statistical Analysis

All assays were performed with platelets from at least three different donors in triplicates. Results are presented as the mean ± standard deviation of representative experiments. Statistical analyses were performed with GraphPad Prism 7 software, using one-way ANOVA with Dunnett's multiple comparison test to check statistically significant values relative to controls. In addition, also unpaired *t*-tests were done to evaluate significant differences between two specified groups. Values of *p* < 0.05 are considered as statistically significant.

## Results

### GAG Deposition on Platelet Surface Induces Opsonization of the Platelets by Complement

**Figure 1:**
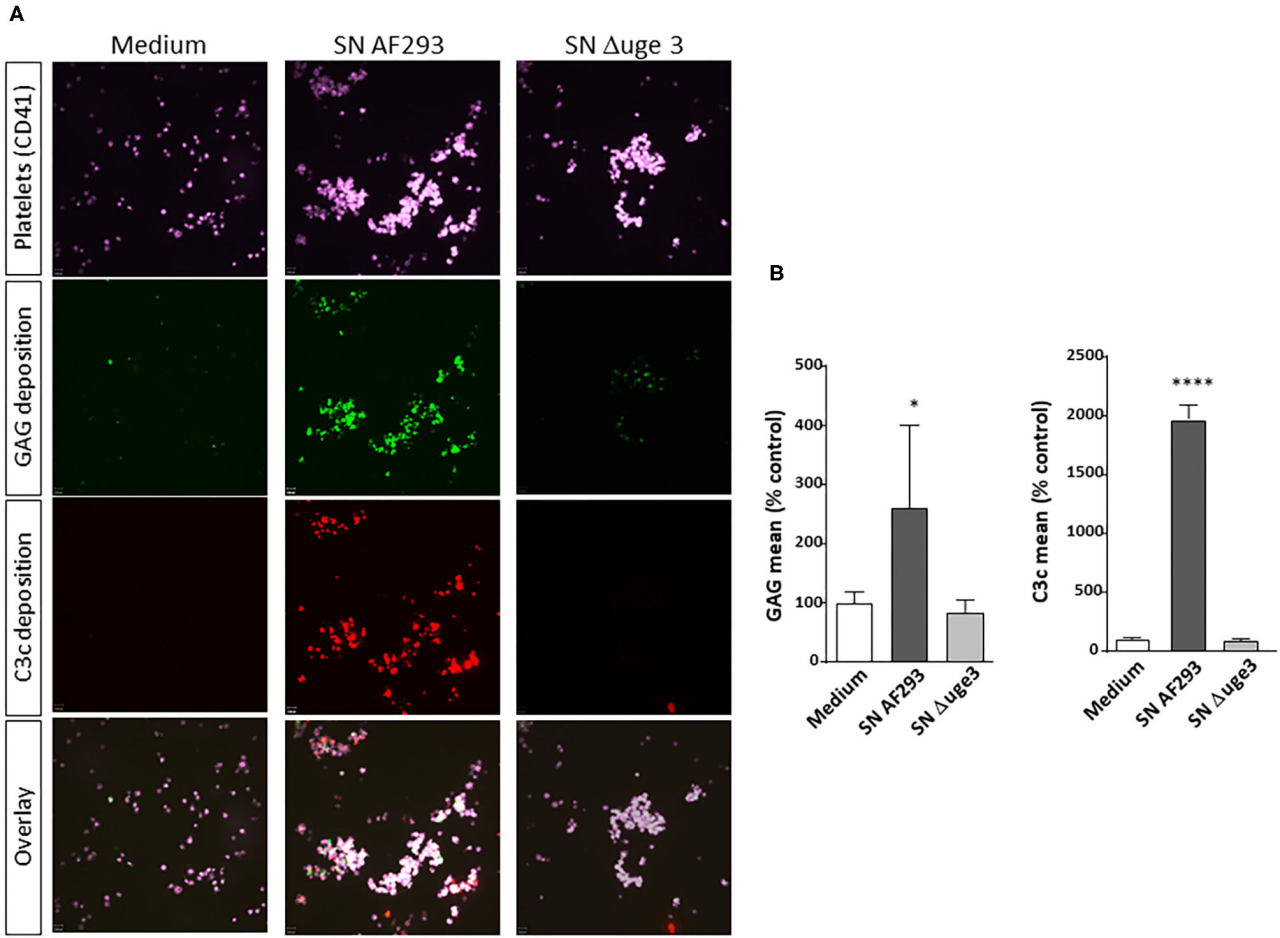
GAG-dependent complement deposition on platelets. Platelets, labeled with a fluorescent αCD41 antibody (purple), were pre-incubated with medium or with 20% supernatant (SN) of *A. fumigatus* wild-type strain (AF293), or the GAG mutant Δ*uge3*, followed by addition of serum as complement source. Deposition of GAG was detected using SBA lectin (green), and opsonization of platelets was investigated by using an αC3c antibody (red). Samples were analyzed by confocal microscopy **(A)** and by flow cytometry **(B)**. Results in **(B)** are analyzed in comparison with the medium sample, which was set to 100%. ****p < 0.001, *p < 0.05.

Previous studies demonstrated that GAG was secreted by *A. fumigatus*, deposited on the surface of platelets, and subsequently induced their activation (9). We therefore analyzed whether GAG deposition leads to the recognition of the platelet surface by the complement system as foreign and thus triggers complement activation. Platelets were incubated with fungal culture SN derived from *A. fumigatus* strain AF293 and, as control, from the GAG-deficient mutant strain Δ*uge3*. GAG deposition on platelets and deposition of complement factor C3 fragments as marker for complement activation were quantified in parallel (Figure 1). Fluorescence microscopy clearly revealed that deposition of GAG and C3c on the platelet surface both occurred with the SN of AF293, whereas SN of the Δ*uge3* mutant induced neither of these processes (Figure 1A). Flow cytometry confirmed and quantified these results; whereas the SN of the Δ*uge3* mutant induced the same background signal for GAG and C3c as medium, the incubation of platelets with SN of AF293 resulted in significant deposition of both GAG and C3c (Figure 1B).

**Figure 2:**
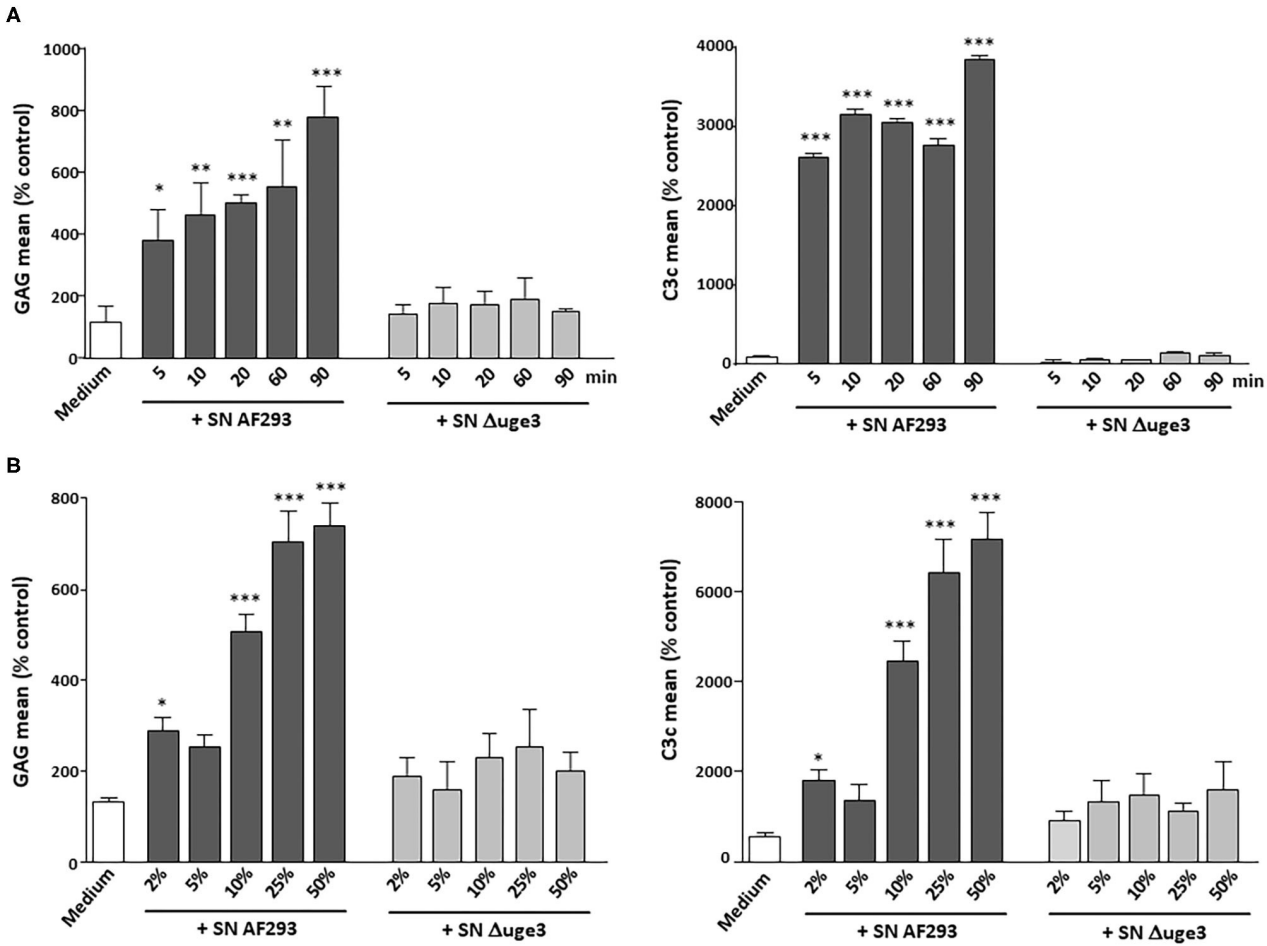
Time and dose curve of GAG-dependent complement deposition on platelets. **(A)** Platelets were incubated for 5–90 min with serum as complement source in the presence of either medium, 20% supernatant (SN) of AF293, or 20% SN of Δ*uge3*. **(B)** Platelets were incubated for 90 min with serum as complement source in the presence of 2–50% SN of AF293 or Δ*uge3*. GAG deposition and opsonization were evaluated by flow cytometry. Each experiment was repeated at least three times with different platelet donors and with triplicate samples. Results are analyzed in comparison with the medium sample, which was set to 100%. ***p < 0.005, **p < 0.01, *p < 0.05.

In addition, time kinetics of GAG deposition on platelets and opsonization by C3 fragments were evaluated and compared. GAG deposition on platelets was a very fast process with a significant signal already 5 min after the start of incubation (Figure 2A). Dose dependence was measured by incubating the platelets with 2–50% SN of AF293 (Figure 2B). A small increase of the GAG signal and of opsonization was visible already with 2% SN. A clear deposition with GAG and opsonization could be achieved with 10% SN. Incubation with SN derived from the Δ*uge3* mutant did not induce any effect, not even at the longest incubation time or at the highest concentration.

**Figure 3:**
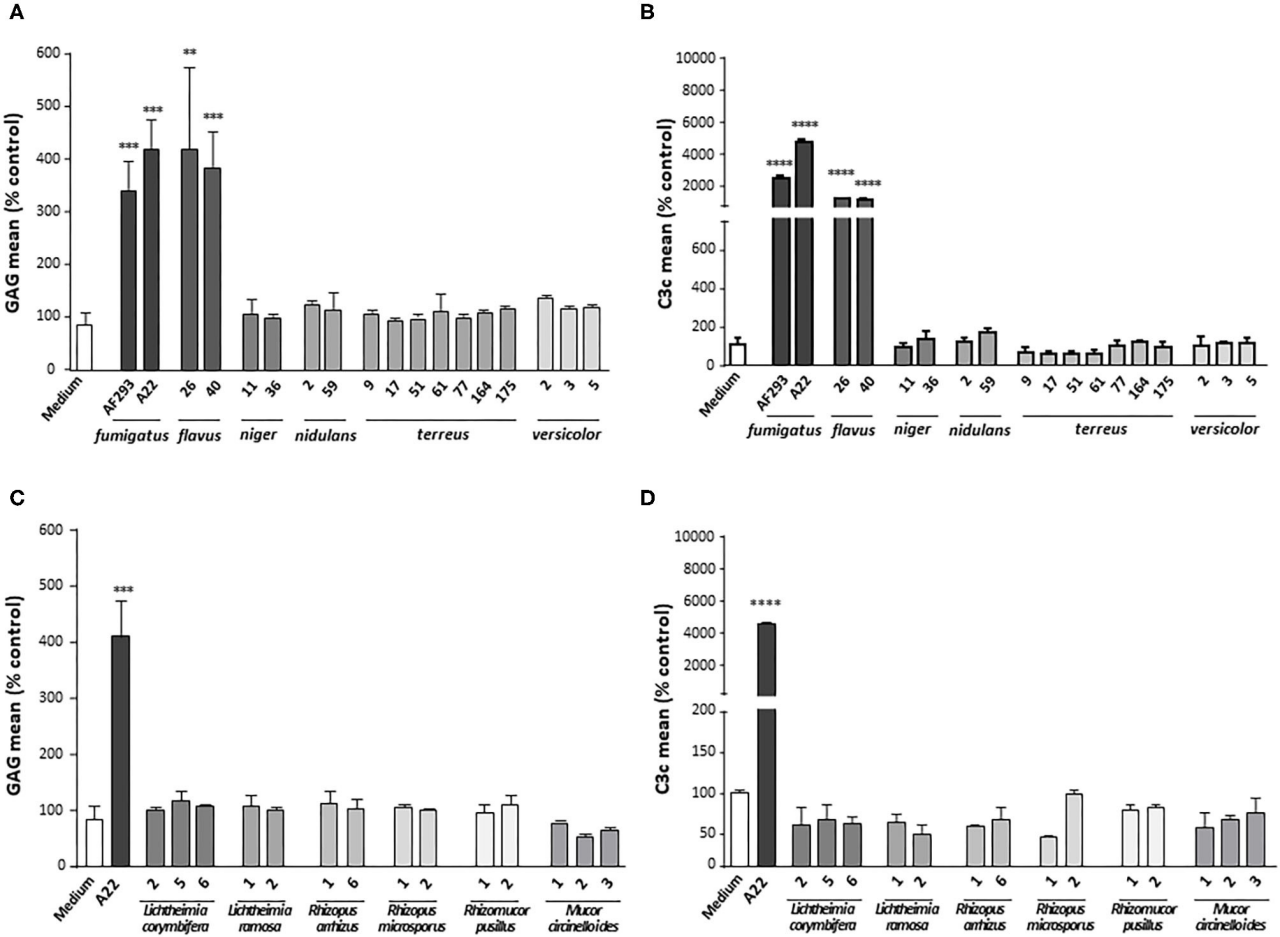
Complement deposition on platelets incubated with different fungal supernatants. Platelets were pre-treated with medium or supernatants (SN) of different isolates of *Aspergillus* species **(A,B)** or Mucorales species **(C,D)**, followed by addition of serum as complement source. GAG deposition **(A,C)** and opsonization **(B,D)** of platelets were quantified by flow cytometry using SBA lectin or an αC3c antibody, respectively. Each experiment was repeated at least three times with different platelet donors and with triplicate samples. Results were shown and statistically analyzed in comparison to medium as a negative control, which was set to 100%. ****p < 0.001, ***p < 0.005, **p < 0.01.

To strengthen the correlation between deposition of GAG on platelets and their opsonization by complement, further SN derived from other fungal species were included in the tests. SN from the clinical *A. fumigatus* isolate A22 as well as from two *A. flavus* strains that all had previously been shown to induce GAG deposition (9) could be demonstrated to also trigger opsonization with C3c on the platelet surface. All other Aspergillus species (*A. niger, A. nidulans, A. terreus*, and *A. versicolor*), negative for GAG secretion, had no effect on C3c deposition (Figures 3A,B). Similarly, the SN of the tested GAG-negative Mucorales species (*Lichtheimia corymbifera, L. ramosa, Rhizopus arrhizus, R. microsporus, Rhizomucor pusillus, and Mucor circinelloides*) lacked the capacity to induce complement deposition on the platelets (Figures 3C,D).

### Relevant Mechanisms of GAG-Induced Platelet Opsonization

**Figure 4:**
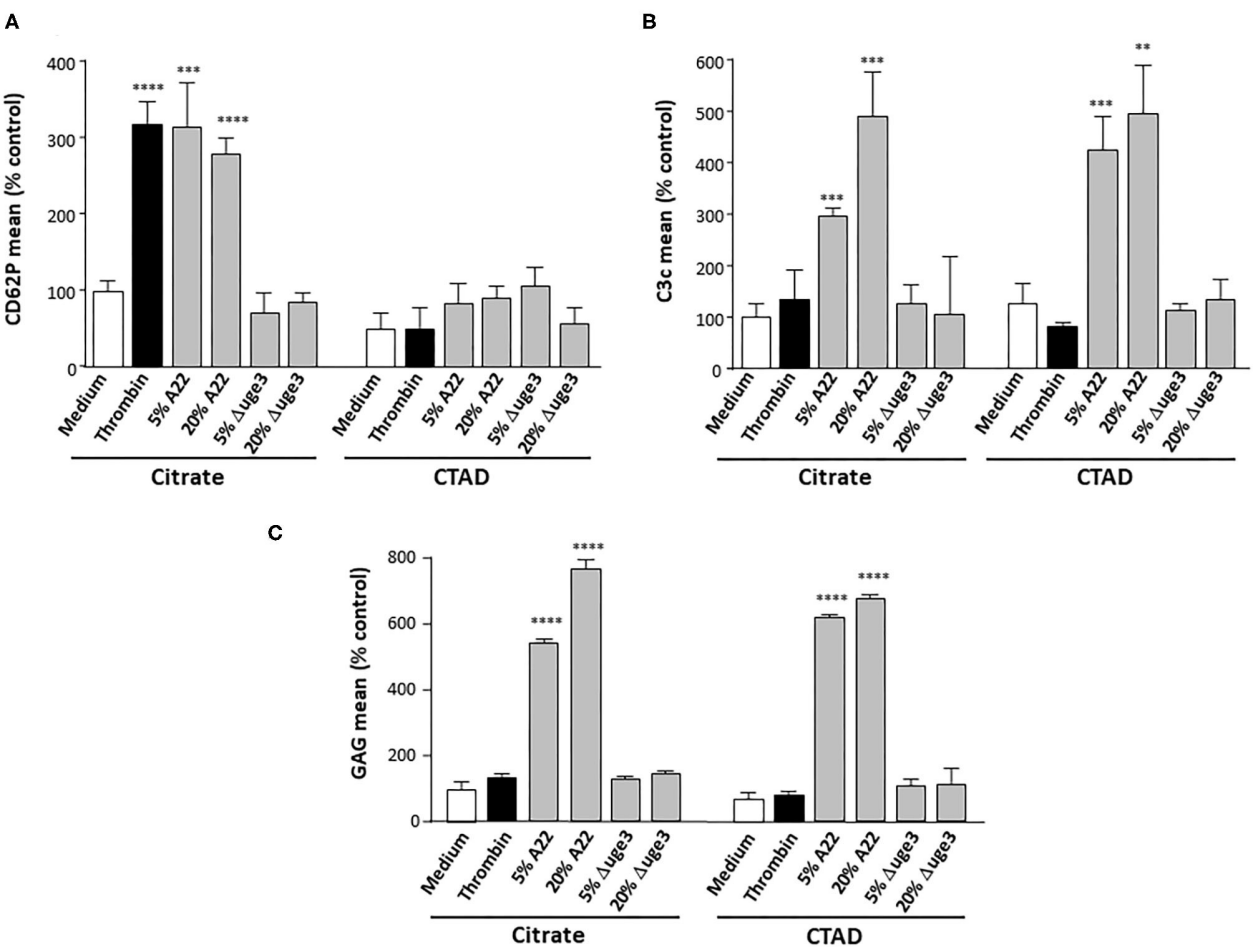
Evaluation of a necessity of platelet activation for GAG-induced platelet opsonization. Blood samples were taken using either citrate or CTAD as anticoagulants, and platelets were isolated. Platelets were pre-treated with medium, thrombin, or 5–20% of supernatant (SN) of *Aspergillus fumigatus* A22 or the Δ*uge3* mutant, followed by addition of serum as complement source. Platelet activation and opsonization were quantified by flow cytometry using an αCD62P antibody as activation marker **(A)** or an αC3c antibody **(B)** to quantify opsonization. Deposition of GAG on the platelets was controlled using SBA lectin **(C)**. Each experiment was repeated at least three times with different platelet donors and with triplicate samples. Results were shown and statistically analyzed in comparison to medium as a negative control, which was set to 100%. ****p < 0.001, ***p < 0.005, **p < 0.01.

In a next step, we aimed to differentiate whether the complement system is triggered by GAG deposition itself or by GAG-induced platelet activation with exposure of phosphatidylserine on the outer leaflet of the plasma membrane (9). For that purpose, platelets were stimulated with thrombin, a well-known strong platelet activator. Whereas, incubation with the fungal SN increased both the activation marker CD62P and the opsonization marker C3c on the platelets, thrombin stimulated only platelet activation but not opsonization (Figures 4A,B). The SN derived from the Δ*uge3* mutant did alter activation or C3c deposition. In addition, CTAD was tested as an alternative anticoagulant for platelet isolation; in contrast to the routinely used citrate, CTAD is known to inhibit platelet activation. Usage of CTAD as an anticoagulant interfered with the SN-induced platelet activation but still allowed the opsonization of platelets with complement fragments (Figures 4A,B). The deposition of GAG on the platelets was similar with both anticoagulants (Figure 4C). These results imply that platelet activation is not a prerequisite for GAG-induced opsonization, and complement recognizes the GAG sheath itself as foreign rather than the GAG-induced phosphatidylserine exposure.

**Figure 5:**
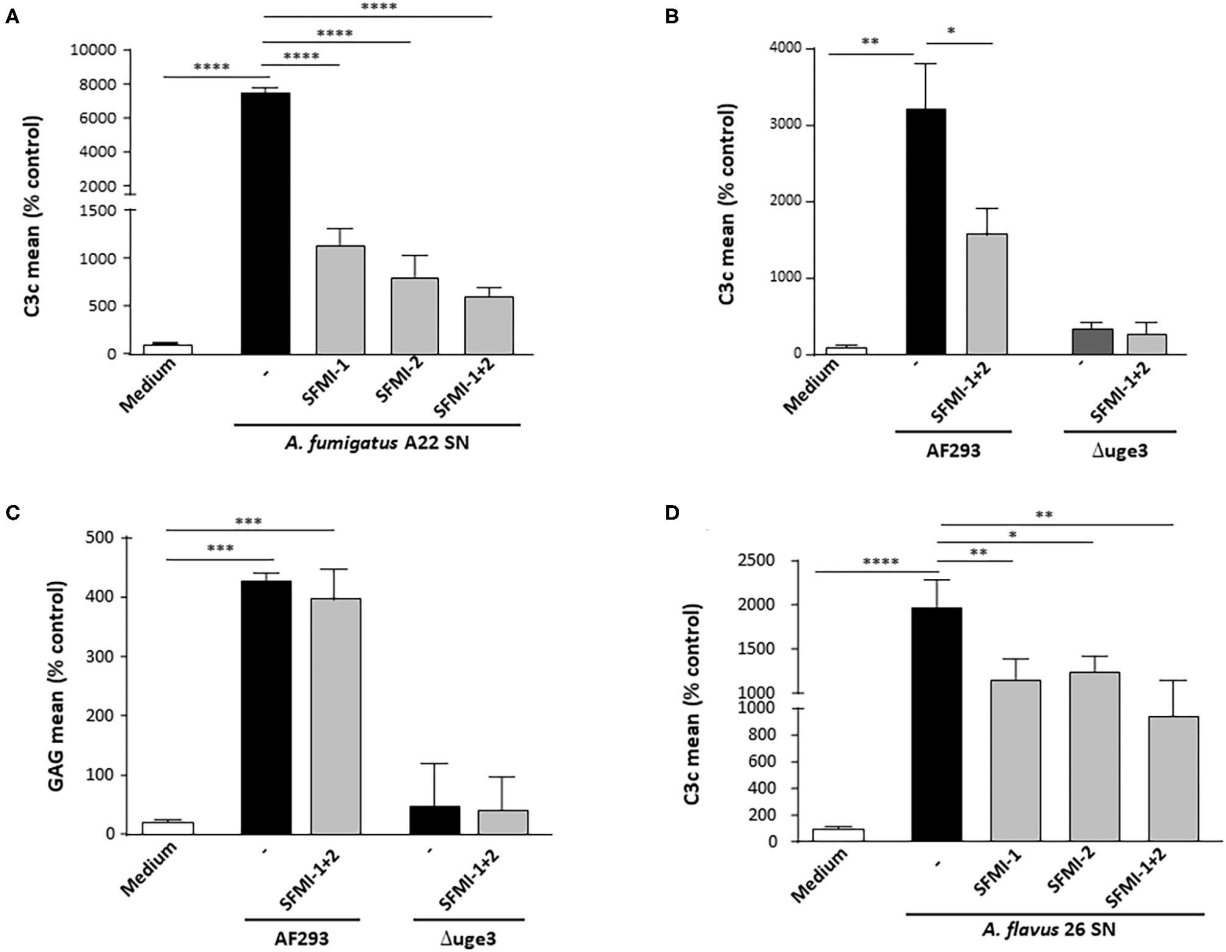
Contribution of lectin pathway to GAG-induced platelet opsonization. Platelets were pre-incubated with medium or 20% supernatant (SN) of *A. fumigatus* A22 **(A)**, *AF293*, and the GAG mutant Δ*uge3* **(B,C)** or *A. flavus* 26 **(D)**. Serum as complement source was added, supplemented with buffer (–) or with the lectin-inhibiting peptides SFMI-1, SFMI-2, or the combination thereof (SFMI-1 + SFMI-2). Complement deposition **(A,B,D)** and deposition of GAG (**C**) on platelets was measured by flow cytometry using an αC3c antibody or SBA lectin, respectively. Results are shown in comparison to medium as a negative control, which was set to 100%. Each experiment was repeated at least three times with different platelet donors and with triplicate samples. ****p < 0.001, ***p < 0.005, **p < 0.01, *p < 0.05.

To get deeper insight into the mechanism of GAG-induced complement activation, we aimed to identify the involved activation pathways. Serum as complement source was supplemented with either EDTA or EGTA before incubation with the platelets and the fungal SN. EDTA blocked all three activation pathways and completely abolished all GAG-triggered C3c deposition on the platelets. EGTA, which blocks the classical and lectin pathway, had a similar effect, indicating that the alternative pathway plays only a minor role (Supplementary Figure 1). Since GAG is a polysaccharide, we hypothesized that the lectin pathway might be the more likely candidate than the classical pathway. To investigate this thesis, we used synthetic peptides that specifically inhibit the lectin pathway by preventing the binding of ficolins or MBL to MASPs. The peptide SFMI-1 inhibits both MASP-1 and MASP-2 binding, whereas peptide SFMI-2 is more potent to inhibit binding of MASP-2. Addition of one of the peptides to the serum as complement source induced a significant reduction of GAG-triggered platelet opsonization, and the effect was even more pronounced when both peptides were added (Figure 5A). This effect was confirmed with SN of the *A. fumigatus* wild-type strain AF293, whereas the C3c signal was negative for the SN of the GAG mutant Δ*uge3*, irrespective of presence or absence of the peptides (Figure 5B). The deposition of GAG was not affected by the peptides (Figure 5C). Since *A. flavus* was the only non-*fumigatus* species that secreted GAG and triggered platelet opsonization, the *A. flavus* SN was also tested with the peptides. Again, the peptides at least partly interfered with the opsonization process, and the effect was most profound when both peptides were added to the serum (Figure 5D). These results indicate that the lectin pathway might play a dominant role in GAG-induced complement opsonization of the platelet surface.

### GAG-Induced Complement Activation on Platelets Triggers Inflammation

**Figure 6:**
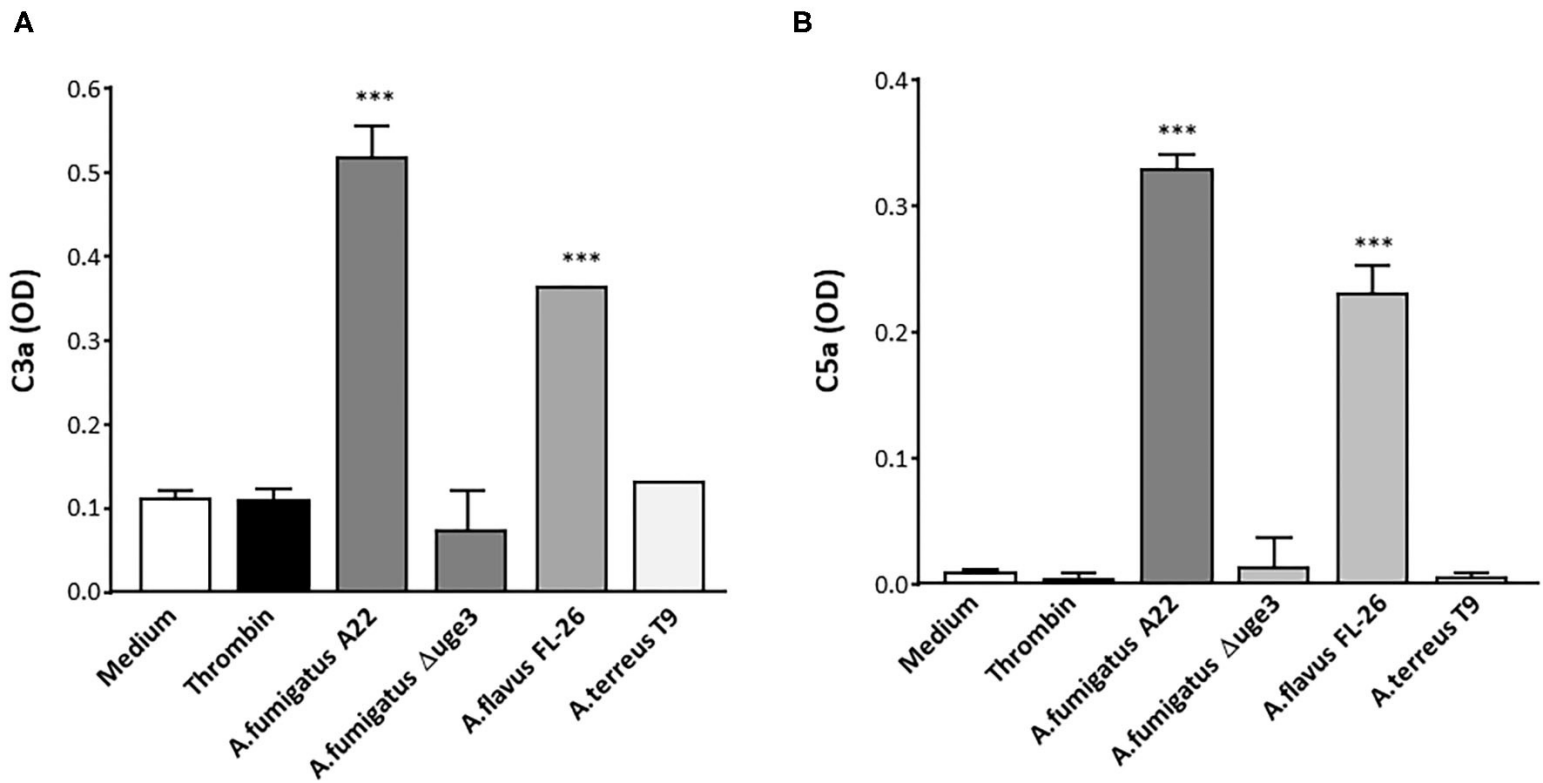
Generation of anaphylatoxins C3a and C5a by GAG-triggered complement activation. Platelets were pre-incubated with medium, thrombin, or 20% supernatant (SN) of *A. fumigatus* (isolate A22), Δ*uge3* mutant *A*. *flavus* (isolate FL-26), or *A. terreus* (isolate T9), followed by addition of serum as complement source. Formation of C3a **(A)** and C5a **(B)** was quantified by ELISA and is given as optical density (OD). Each experiment was repeated at least three times with different platelet donors and with triplicate samples. Results were statistically analyzed in comparison to medium as a negative control. ***p < 0.005.

To monitor the physiological relevance of GAG-induced complement activation on platelets, formation of the complement factors C3a and C5a after contact of platelets with fungal SN was quantified. These pro-inflammatory anaphylatoxins are putative mediators of thrombosis and inflammation (20, 21) and thus might essentially contribute to these hallmarks of invasive fungal infections. Respective ELISAs demonstrated a significantly higher formation of both anaphylatoxins when platelets were incubated in the presence of serum with SN of *A. fumigatus* and *A. flavus* than in medium or thrombin controls (Figures 6A,B). In contrast, the SN of *A. terreus* or the Δ*uge3* mutant, which were not able to produce or secrete GAG, did not induce generation of C3a or C5a.

**Figure 7:**
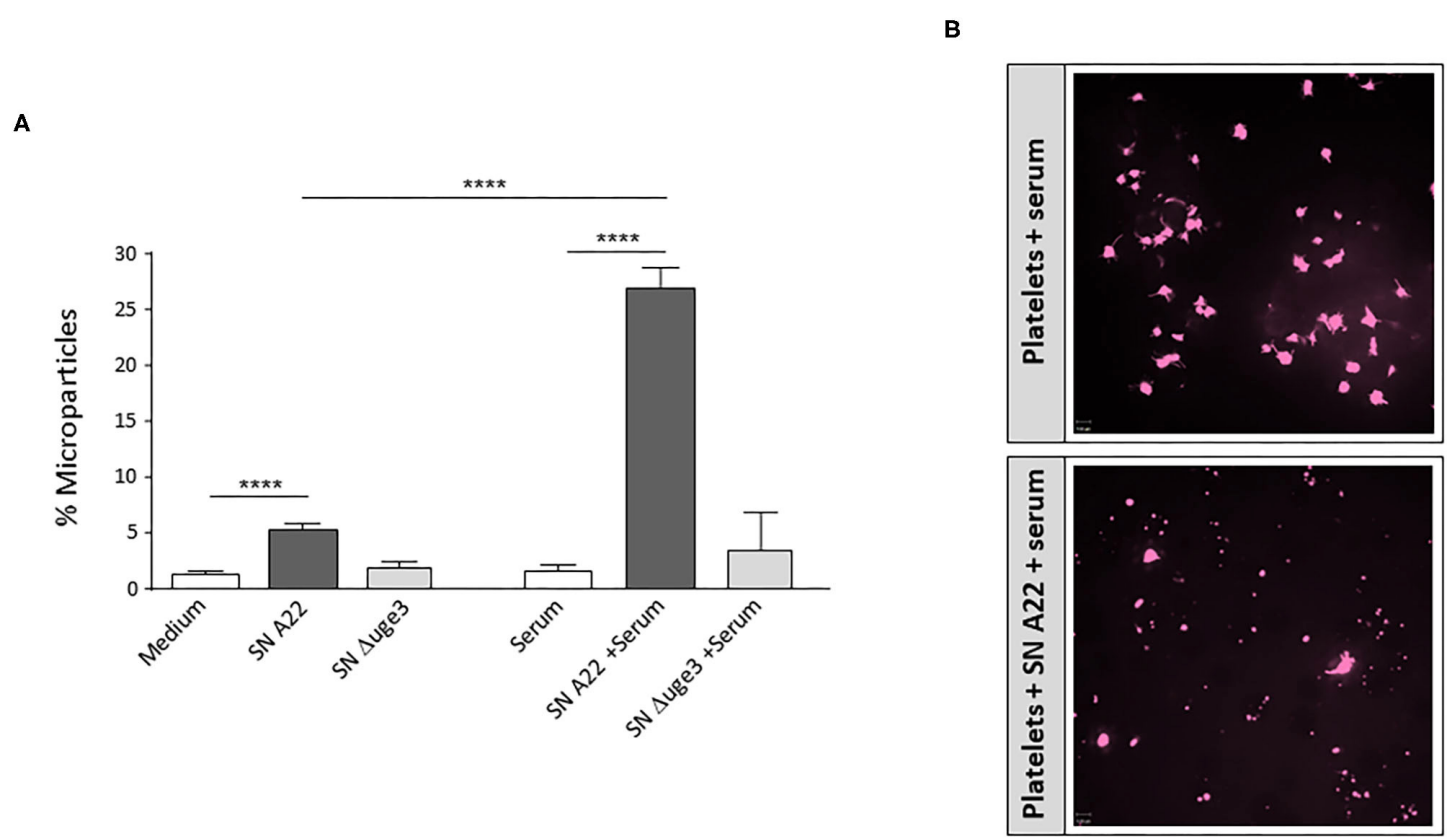
Formation of microparticles upon GAG-induced platelet opsonization. **(A)** Platelets, labeled with a fluorescent αCD41 antibody, were incubated with medium, 20% supernatant (SN) of *A. fumigatus* strain A22 or Δ*uge3* mutant, serum, or 20% fungal supernatants plus serum. Newly formed microparticles were gated in flow cytometry according to their size and are expressed as percentage of all CD41-positive events. **(B)** Microparticle formation was also visualized by confocal microscopy. Each experiment was repeated at least three times with different platelet donors and with triplicate samples. ****p < 0.001.

Budding of microparticles is another mechanism how platelets can contribute to inflammation, since they can contribute to dissemination of pro-inflammatory signals (cytokines and chemokines) (22). We evaluated whether GAG-induced platelet opsonization might trigger microparticle formation. As described previously, incubation of platelets with SN of GAG-secreting *A. fumigatus* enhanced the percentage of microparticles (Figure 7A). When incubation of platelets with SN was performed in the presence of serum, the percentage of microparticles was five times higher than with SN alone (Figure 7A). Confocal microscopy confirmed the enhanced SN-induced microparticle formation in the presence of serum (Figure 7B). In contrast to the wild type, SN of the Δ*uge3* mutant did not significantly alter the percentage of microparticles compared to the medium control (Figure 7A).

### GAG-Induced Complement Activation on Platelets Triggers Platelet Loss

**Figure 8:**
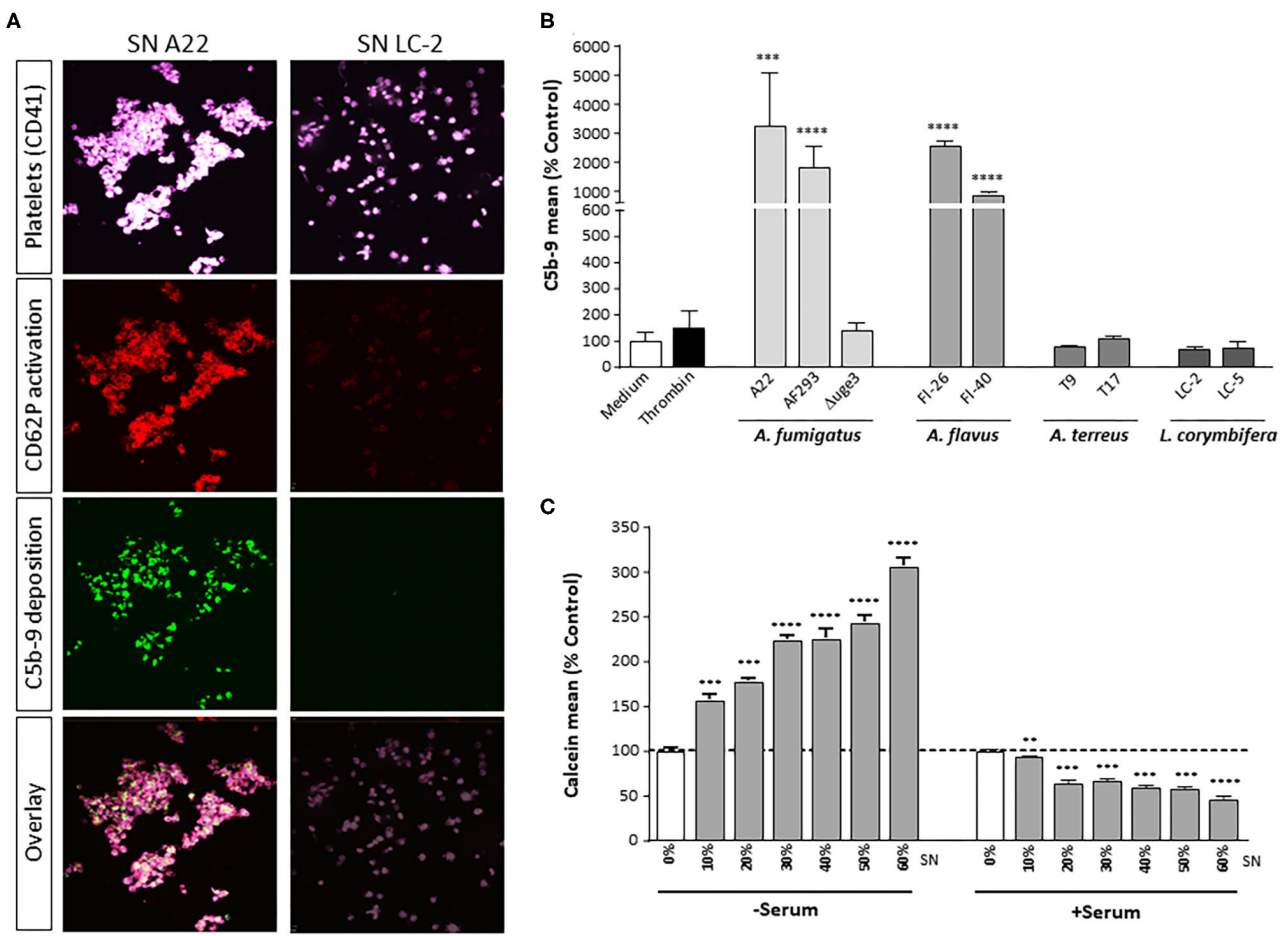
GAG-dependent complement C5b-9 formation on platelets and platelet viability. **(A,B)** Platelets, labeled with a fluorescent αCD41 antibody (purple), were pre-incubated with medium or with 20% supernatant (SN) of different fungal species, followed by addition of serum as complement source. Activation of platelets was monitored **(A)** by a specific αCD62P antibody. Generation of C5b-9 on the platelet surface was detected by confocal microscopy **(A)** or quantified by flow cytometry **(B)**. **(C)** Platelets were pre-incubated with increasing concentrations of *A. fumigatus* A22 supernatant (SN), followed by addition of medium or serum as complement source. Platelet viability was monitored by addition of calcein blue and analyzed by flow cytometry. Each experiment was repeated at least three times with different platelet donors and with triplicate samples. Results in **(B,C)** are analyzed in comparison with the medium sample, which was set to 100%. ****p < 0.001, ***p < 0.005, **p < 0.01.

The complement cascade ends in the terminal pathway with formation of the C5b-9 complex. Whereas, the soluble form (TCC) contributes to immune cell activation, the membrane-bound form (MAC) forms a pore to induce membrane disintegration and lysis. We therefore monitored whether secreted GAG leads to C5b-9 formation on the platelet surface. Incubation of platelets with fungal SN of *A. fumigatus* A22 triggered formation of the C5b-9 complex on the surface, whereas the signal was negative with SN of *Lichtheimia corymbifera* LC-2 (Figure 8A). A broad screening and quantitative analysis of different isolates and species confirmed that GAG-secreting isolates of *A. fumigatus* and *A. flavus* all induced C5b-9 formation on platelets, while species without GAG secretion, such as *A. terreus L. corymbifera*, or the Δ*uge3* mutant, did not (Figure 8B).

GAG-induced formation of C5b-9 could also be correlated with loss of platelet viability. Platelets were incubated with increasing concentrations of SN of *A. fumigatus* A22, in the presence or absence of serum as complement source. Calcein blue, a cell-permeant esterase substrate, serves as a viability probe that measures both activity of intracellular esterases and cell-membrane integrity. Incubation of platelets with fungal SN alone results in a concentration-dependent staining intensity of platelets with calcein blue, reflecting the SN-induced platelet activation (Figure 8C). In the presence of serum as complement source, however, the calcein blue signal decreases significantly, again in a concentration-dependent manner (Figure 8C). Addition of heat-inactivated serum did not affect staining with calcein blue and thereby platelet viability (data not shown).

## Discussion

The angioinvasive capacity of human-pathogenic *Aspergillus* species makes complement and platelets prevailing targets of interest. Both are elements of innate immunity, and both are present in the blood in high concentrations/amounts. The present study revealed that deposition of fungal GAG on the surface of platelets results in activation of the complement cascade with respective consequences of complement activity. Fungal secretory products such as GAG are commonly supposed to play a substantial role in the pathogenesis of invasive fungal infections (23). Their action is not limited to the site of infection and the presence of fungal hyphae, but they can easily disseminate in the bloodstream and exert their impact throughout the body and distant from the focus of infection. In the stage of hematogenic spreading of the invading fungi, the high concentration of platelets in the blood draws the attention on the effect of secretory compounds on platelets.

The surface-bound polysaccharide GAG, produced by *A. fumigatus* and *A. flavus*, was previously shown to mediate the interaction between hyphae and platelets (24). The spectrum of GAG–platelet interactions was expanded by findings that also secreted GAG affects platelets by binding to their surface and inducing their activation (9). In the experiments presented here, we could allocate a further function to GAG, i.e., the triggering of a complement reaction against platelets.

The close relationship between complement and platelets includes multiple axes and molecular mechanisms of mutual regulation [reviewed in (25, 26)]. Upon activation, platelets were described to expose P-selectin (CD62P) on their surface, which subsequently triggers the alternative complement pathway (27, 28). This is in contrast to our findings, which indicate that thrombin-induced activation alone is not sufficient to induce significant complement activation. Possible explanations of this discrepancy might be that these research groups used other platelet activators such as TRAP, ADP, or arachidonic acid. Only one experiment used thrombin as activator, but at 10-fold higher concentrations than in our assays (1 IU/ml vs. 0.1 IU/ml, respectively).

Furthermore, in our experiments, CTAD, which is an alternative anticoagulant inhibiting platelet activation and CD62P exposition, still enabled GAG-induced complement deposition on the platelet surface. According to this finding, the deposition of fungal GAG on platelets appears to be the main trigger of complement activation in our experimental setting. This thesis was strengthened by experiments applying lectin pathway-inhibiting peptides. Whereas, CD62P exposition was described to mainly initiate the alternative pathway (27, 28), inhibition of the lectin pathway by these peptides significantly interfered with GAG-induced complement activation. This finding is consistent with the fact that GAG is a polysaccharide and therefore a natural target for the lectin pathway specialized on the recognition of microbial carbohydrates. The relevance of the lectin pathway for GAG-stimulated complement activation might also partly explain why MBL-deficient mice showed an improved survival in a mouse model of systemic aspergillosis, as reviewed elsewhere (29).

Data of mouse models indicate that platelets represent a key factor to maintain hemostasis and lung integrity in response to exposed fungal antigens. Thrombocytopenic mice exhibited severe hemorrhage into the airways in response to *A. fumigatus* challenge (30). Since platelets are versatile antifungal immune weapons (10, 11), their shutoff via the body's own complement cascade represents a perfidious mechanism of fungal immune evasion. Indications that elimination of platelet immunology affects the prognosis of infected patients came from studies showing that low baseline platelet counts predict a poor outcome of invasive aspergillosis (31). Lowered platelet count in invasive fungal disease was also described in preterm infants, and antifungal treatment significantly elevated the platelet number in blood (32). The consequence of GAG-triggered complement attack against platelets is the loss of platelet viability and, as a result, the loss of platelet-associated immune and hemostatic capacities. Platelet loss might be reinforced by interaction with neutrophils that may bind via their complement receptors to opsonized platelets and may trigger their phagocytosis and destruction.

GAG-mediated complement activation on the platelet surface might not only damage the platelets themselves but also affect “bystander” cells, thus contributing to cell loss during invasive aspergillosis. Tissue damage mediated by activated complement (“friendly fire”) is evident in numerous diseases, including arthritis, cancer, stroke, and infections (33, 34). Further studies, e.g., by co-incubation of platelets and GAG with putative bystander cells, will help to confirm the relevance of this mechanism.

GAG-driven complement activation might not only induce platelet loss, thrombocytopenia, and bystander lysis in infected patients but also participate in thrombosis and inflammation. Our results show that significant amounts of complement-derived anaphylatoxins C3a and C5a are generated after triggering GAG deposition on platelets in the presence of serum as complement source. In addition, platelets were shown to bear receptors for C3a and C5a (35, 36) themselves. Binding of the generated anaphylatoxins to their receptors on circulating “bystander” platelets may further enhance the participation of platelets in thrombotic and inflammatory processes (26, 36). Furthermore, C5a was described to cause profound prothrombotic perturbations on the endothelium and thus to drive macrovascular thrombosis (37). Released anaphylatoxins might also chemotactically attract other immune cells such as neutrophils and thus contribute to initiation of pro-inflammatory processes (38).

According to our results, a further amplification of thrombus formation might be provoked by GAG-driven shedding of pro-coagulant platelet microparticles. Microparticle formation might be stimulated by C5a or C5b-9 formation, which both were detected after incubation of platelets with GAG-containing fungal SN and subsequent presence of serum. These complement activation products were described to promote the shedding of microparticles (37). The released microparticles budding from the plasma membrane of platelets expose phospholipids on their surface that act as procoagulants (39). Platelet-derived microparticles are even reported to have a 50- to 100-fold higher procoagulant activity than activated platelets (40). Thus, secretion of fungal GAG with its subsequent effects on platelet-complement interaction might be a serious perpetrator for thrombosis in aspergillosis patients (41), predominantly in those with predisposing factors such as prolonged neutropenia, chronic administration of corticosteroids, insertion of prosthetic devices, or tissue damage due to prior infection or trauma (42).

Both GAG-induced anaphylatoxins and microparticles also act in a pro-inflammatory manner and thereby might mediate excessive inflammation during invasive aspergillosis. Microparticles contain chemokines (e.g., RANTES) and cytokines (e.g., IL-1β) and thus are able to trigger inflammation distant from the site of infection (43). C5a is a well-known chemoattractant and recruits immune cells into inflamed tissue (44). C3a is mainly described as a pro-inflammatory molecule but also shows several anti-inflammatory facets (20).

Interestingly, all tested Mucorales species were unable to secrete GAG, and their culture SN did not induce activation of platelets or complement activation on the platelet surface. This is surprising, since angioinvasion and thrombosis are typical hallmarks of invasive mucormycosis, as reviewed elsewhere (45). Our results, however, indicate that the mechanism of Mucorales-induced thrombosis does not involve a pro-thrombotic activity of a secreted compound.

In summary, GAG deposition on platelets leads to complement activation and might thus support the attack against the invading fungal pathogens. The activated complement might also trigger other arms of innate and adaptive immune system. However, putative negative consequences are thrombocytopenia, thrombosis, and excessive inflammation. *In vivo* studies in a mouse model will help to assess the influence of GAG on the complement–platelet axis during invasive aspergillosis.

## Data Availability Statement

The raw data supporting the conclusions of this article will be made available by the authors, without undue reservation.

## Ethics Statement

The studies involving human participants were reviewed and approved by Ethics committee of the Medical University of Innsbruck. The patients/participants provided their written informed consent to participate in this study.

## Author Contributions

CS, GR, DS, RW, and CL-F contributed to the conception and design of the study. HD and MN performed experiments. CS and MN made statistical analysis. CS, HD, and GR wrote sections of the manuscript. All authors contributed to the revision of the manuscript, read, and approved the submitted version.

## Conflict of Interest

The authors declare that the research was conducted in the absence of any commercial or financial relationships that could be construed as a potential conflict of interest.

## References

[1] Mellinghoff SC Panse J Alakel N Behre G Buchheidt D Christopeit M. Primary prophylaxis of invasive fungal infections in patients with haematological malignancies: 2017 update of the recommendations of the Infectious Diseases Working Party (AGIHO) of the German Society for Haematology and Medical Oncology (DGHO). Ann Hematol. (2018) 97:197–207. 10.1007/s00277-017-3196-2 29218389 PMC5754425

[2] Otto WR Green AM. Fungal infections in children with haematologic malignancies and stem cell transplant recipients. Br J Haematol. (2020) 189:607–24. 10.1111/bjh.16452 32159231 PMC7231650

[3] Latge JP Chamilos G. Aspergillus fumigatus and Aspergillosis in 2019. Clin Microbiol Rev Dec. (2019) 18:33. 10.1128/CMR.00140-18 31722890 PMC6860006

[4] Lehrnbecher T Frank C Engels K Kriener S Groll AH Schwabe D. Trends in the postmortem epidemiology of invasive fungal infections at a university hospital. J Infect. (2010) 61:259–65. 10.1016/j.jinf.2010.06.018 20624423

[5] Subira M Martino R Rovira M Vazquez L Serrano D De la Camara R. Clinical applicability of the new EORTC/MSG classification for invasive pulmonary aspergillosis in patients with hematological malignancies and autopsy-confirmed invasive aspergillosis. Ann Hematol. (2003) 82:80–2. 10.1007/s00277-002-0599-4 12601484

[6] Brown GD Denning DW Gow NA Levitz SM Netea MG White TC. Hidden killers: human fungal infections. Sci Transl Med. (2012) 4:165rv113. 10.1126/scitranslmed.3004404 23253612

[7] Lee MJ Liu H Barker BM Snarr BD Gravelat FN Al Abdallah Q. The fungal exopolysaccharide galactosaminogalactan mediates virulence by enhancing resistance to neutrophil extracellular traps. PLoS Pathog. (2015) 11:e1005187. 10.1371/journal.ppat.1005187 26492565 PMC4619649

[8] Speth C Rambach G Lass-Florl C Howell PL Sheppard DC. Galactosaminogalactan (GAG) and its multiple roles in *Aspergillus* pathogenesis. Virulence. (2019) 22:1–8. 10.1080/21505594.2019.1568174 30667338 PMC8647848

[9] Deshmukh H Rambach G Sheppard DC Lee M Hagleitner M Hermann M. Galactosaminogalactan secreted from *Aspergillus fumigatus* and *Aspergillus flavus* induces platelet activation. Microbes Infect. (2020) 18:4. 10.1016/j.micinf.2019.12.004 31962135

[10] Speth C Loffler J Krappmann S Lass-Florl C Rambach G. Platelets as immune cells in infectious diseases. Future Microbiol. (2013) 8:1431–51. 10.2217/fmb.13.104 24199802

[11] Speth C Rambach G Lass-Florl C. Platelet immunology in fungal infections. Thromb Haemost. (2014) 112:632–9. 10.1160/TH14-01-0074 24990293

[12] McDonald B Dunbar M. Platelets and intravascular immunity: guardians of the vascular space during bloodstream infections and sepsis. Front Immunol. (2019) 10:2400. 10.3389/fimmu.2019.02400 31681291 PMC6797619

[13] Kumar V. The complement system, toll-like receptors and inflammasomes in host defense: three musketeers' one target. Int Rev Immunol. (2019) 38:131–56. 10.1080/08830185.2019.1609962 31066339

[14] Mongini PK Jackson AE Tolani S Fattah RJ Inman JK. Role of complement-binding CD21/CD19/CD81 in enhancing human B cell protection from Fas-mediated apoptosis. J Immunol. (2003) 171:5244–54. 10.4049/jimmunol.171.10.5244 14607925

[15] Ajona D Ortiz-Espinosa S Pio R. Complement anaphylatoxins C3a and C5a: Emerging roles in cancer progression and treatment. Semin Cell Dev Biol. (2019) 85:153–63. 10.1016/j.semcdb.2017.11.023 29155219

[16] Xie CB Jane-Wit D Pober JS. Complement membrane attack complex: new roles, mechanisms of action, and therapeutic targets. Am J Pathol. (2020) 16:6. 10.1016/j.ajpath.2020.02.006 32194049 PMC7280757

[17] Wostemeyer J. (1985). Strain-dependent variation in ribosomal DNA arrangement in Absidia glauca. Eur J Biochem. 146:443–8. 10.1111/j.1432-1033.1985.tb08671.x 2578394

[18] Lee MJ Gravelat FN Cerone RP Baptista SD Campoli PV Choe SI. Overlapping and distinct roles of Aspergillus fumigatus UDP-glucose 4-epimerases in galactose metabolism and the synthesis of galactose-containing cell wall polysaccharides. J Biol Chem. (2014) 289:1243–56. 10.1074/jbc.M113.522516 24257745 PMC3894311

[19] Gravelat FN Beauvais A Liu H Lee MJ Snarr BD Chen D. *Aspergillus galactosaminogalactan* mediates adherence to host constituents and conceals hyphal beta-glucan from the immune system. PLoS Pathog. (2013) 9:e1003575. 10.1371/journal.ppat.1003575 23990787 PMC3749958

[20] Coulthard LG Woodruff TM. Is the complement activation product C3a a proinflammatory molecule? Re-evaluating the evidence and the myth. J Immunol. (2015) 194:3542–8. 10.4049/jimmunol.1403068 25848071

[21] Kourtzelis I Markiewski MM Doumas M Rafail S Kambas K Mitroulis I. Complement anaphylatoxin C5a contributes to hemodialysis-associated thrombosis. Blood. (2010) 116:631–9. 10.1182/blood-2010-01-264051 20424189 PMC3086498

[22] Kapur R Zufferey A Boilard E Semple JW. Nouvelle cuisine: platelets served with inflammation. J Immunol. (2015) 194:5579–87. 10.4049/jimmunol.1500259 26048965

[23] Ghannoum MA. Potential role of phospholipases in virulence and fungal pathogenesis. Clin Microbiol Rev. (2000) 13:122–43. 10.1128/CMR.13.1.122 10627494 PMC88936

[24] Rambach G Blum G Latge JP Fontaine T Heinekamp T Hagleitner M. Identification of *Aspergillus fumigatus* surface components that mediate interaction of conidia and hyphae with human platelets. J Infect Dis. (2015) 212:1140–9. 10.1093/infdis/jiv191 25810442

[25] Eriksson O Mohlin C Nilsson B Ekdahl KN. The human platelet as an innate immune cell: interactions between activated platelets and the complement system. Front Immunol. (2019) 10:1590. 10.3389/fimmu.2019.01590 31354729 PMC6635567

[26] Speth C Rambach G Wurzner R Lass-Florl C Kozarcanin H Hamad OA. Complement and platelets: mutual interference in the immune network. Mol Immunol. (2015) 67:108–18. 10.1016/j.molimm.2015.03.244 25886718

[27] Del Conde I Cruz MA Zhang H Lopez JA Afshar-Kharghan V. Platelet activation leads to activation and propagation of the complement system. J Exp Med. (2005) 201:871–9. 10.1084/jem.20041497 15781579 PMC2213112

[28] Saggu G Cortes C Emch HN Ramirez G Worth RG Ferreira VP. Identification of a novel mode of complement activation on stimulated platelets mediated by properdin and C3(H2O). J Immunol. (2013). 190:6457–67. 10.4049/jimmunol.1300610 23677468 PMC3784323

[29] Parente R Doni A Bottazzi B Garlanda C Inforzato A. The complement system in *Aspergillus fumigatus* infections and its crosstalk with pentraxins. FEBS Lett. (2020) 28:13744. 10.1002/1873-3468.13744 31994174

[30] Tischler BY Tosini NL Cramer RA Hohl TM. Platelets are critical for survival and tissue integrity during murine pulmonary *Aspergillus fumigatus* infection. PLoS Pathog. (2020) 16:e1008544. 10.1371/journal.ppat.1008544 32407390 PMC7252636

[31] Nouer SA Nucci M Kumar NS Grazziutti M Restrepo A Anaissie E. Baseline platelet count and creatinine clearance rate predict the outcome of neutropenia-related invasive aspergillosis. Clin Infect Dis. (2012) 54:e173–83. 10.1093/cid/cis298 22423136 PMC3404713

[32] Zhao D Qiu G Luo Z Zhang Y. Platelet parameters and (1, 3)-beta-D-glucan as a diagnostic and prognostic marker of invasive fungal disease in preterm infants. PLoS ONE. (2015) 10:e0123907. 10.1371/journal.pone.0123907 25874376 PMC4395423

[33] Arumugam TV Woodruff TM Lathia JD Selvaraj PK Mattson MP Taylor SM. Neuroprotection in stroke by complement inhibition and immunoglobulin therapy. Neuroscience. (2009) 158:1074–89. 10.1016/j.neuroscience.2008.07.015 18691639 PMC2639633

[34] Koscielska-Kasprzak K Bartoszek D Myszka M Zabinska M Klinger M. The complement cascade and renal disease. Archiv Immunol Therap Exp. (2014) 62:47–57. 10.1007/s00005-013-0254-x 24030732 PMC3898353

[35] Martel C Cointe S Maurice P Matar S Ghitescu M Theroux P. Requirements for membrane attack complex formation and anaphylatoxins binding to collagen-activated platelets. PLoS ONE. (2011) 6:e18812. 10.1371/journal.pone.0018812 21526204 PMC3078139

[36] Patzelt J Mueller KA Breuning S Karathanos A Schleicher R Seizer P. Expression of anaphylatoxin receptors on platelets in patients with coronary heart disease. Atherosclerosis. (2015) 238:289–95. 10.1016/j.atherosclerosis.2014.12.002 25544179

[37] Noris M Remuzzi G Terminal complement effectors in atypical hemolytic uremic syndrome: C5a, C5b-9, or a bit of both? Kidney Int. (2019) 96:13–5. 10.1016/j.kint.2019.02.038 31229026

[38] Sadik CD Miyabe Y Sezin T Luster AD. The critical role of C5a as an initiator of neutrophil-mediated autoimmune inflammation of the joint and skin. Semin Immunol. (2018) 37:21–9. 10.1016/j.smim.2018.03.002 29602515

[39] Nomura S Shimizu M. Clinical significance of procoagulant microparticles. J Intensive Care. (2015) 3:2. 10.1186/s40560-014-0066-z 25705427 PMC4336124

[40] Sinauridze EI Kireev DA Popenko NY Pichugin AV Panteleev MA Krymskaya OV. Platelet microparticle membranes have 50- to 100-fold higher specific procoagulant activity than activated platelets. Thromb Haemost. (2007) 97:425–34. 10.1160/TH06-06-0313 17334510

[41] Ben-Ami R. Angiogenesis at the mold-host interface: a potential key to understanding and treating invasive aspergillosis. Future Microbiol. (2013) 8:1453–62. 10.2217/fmb.13.114 24199803

[42] Gundlach JP Gunther R Fickenscher H Both M Rocken C Becker T. Lethal thrombosis of the iliac artery caused by *Aspergillus fumigatus* after liver transplantation: case report and review of the literature. BMC Surg. (2019) 19:200. 10.1186/s12893-019-0668-4 31881871 PMC6935117

[43] Koupenova M Clancy L Corkrey HA Freedman JE. Circulating platelets as mediators of immunity, inflammation, and thrombosis. Circ Res. (2018) 122:337–51. 10.1161/CIRCRESAHA.117.310795 29348254 PMC5777300

[44] Zaal A van Ham SM Ten Brinke A. Differential effects of anaphylatoxin C5a on antigen presenting cells, roles for C5aR1 and C5aR2. Immunol Lett. (2019) 209:45–52. 10.1016/j.imlet.2019.03.014 30959077

[45] Ghuman H Voelz K. Innate and adaptive immunity to mucorales. J Fungi. (2017) 3:48. 10.3390/jof3030048 29371565 PMC5715954

