# Supplementary figures and images for: Aspergillus-Derived Galactosaminogalactan Triggers Complement Activation on Human Platelets
Source: Front Immunol. 2020 Oct 6;11:550827. doi: 10.3389/fimmu.2020.550827 (PMC7573070; doi:10.3389/fimmu.2020.550827)

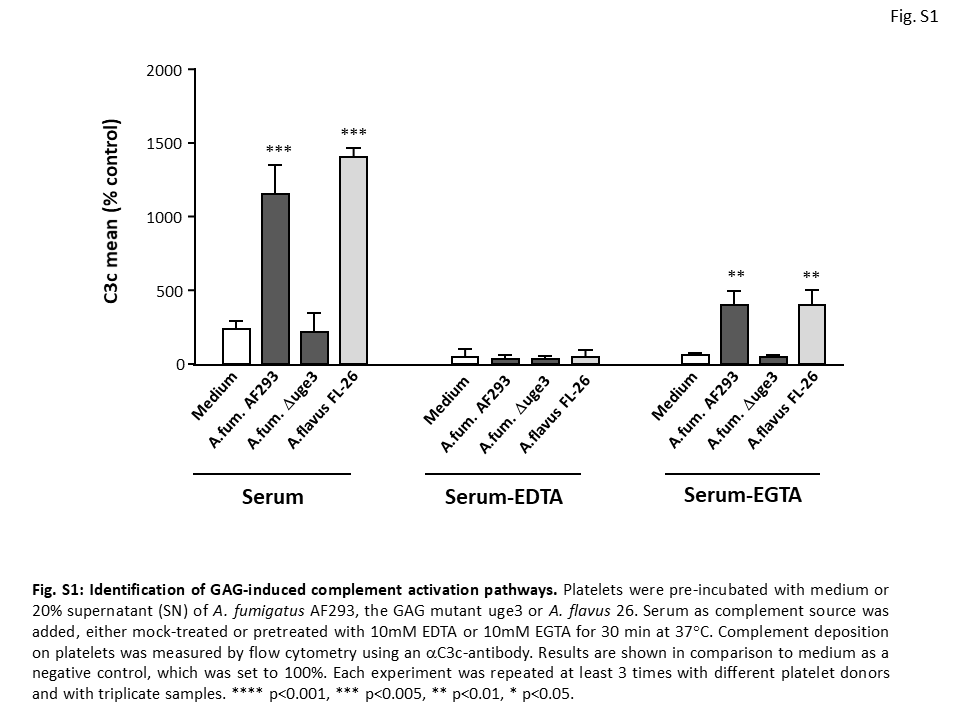

Supplement: Supplementary file 1 [file Image_1.TIF]
